# Stigma against Tuberculosis Patients in Addis Ababa, Ethiopia

**DOI:** 10.1371/journal.pone.0152900

**Published:** 2016-04-07

**Authors:** Sebsibe Tadesse

**Affiliations:** Institute of Public Health, the University of Gondar, Gondar, Ethiopia; University of Missouri-Kansas City, UNITED STATES

## Abstract

**Background:**

Stigma attached to tuberculosis contributes to the limited effectiveness of current TB control approaches. However, there is a dearth of studies that explore the causes of stigma attached to tuberculosis and its effects on patients and tuberculosiscontrol programs in Ethiopia.

**Methods:**

An institution-based qualitative study was conducted at St. Peter Tuberculosis Specialized Hospital in Addis Ababa, Ethiopia from July to August, 2015. Ten in-depth interviews and 6 key-informant interviews were carried out among tuberculosis patients and healthcare workers, respectively.The Open Code computer software package was used to analyze the data thematically.

**Results:**

The study revealed that fear of infection and inappropriate health education messages by media were the main causes of tuberculosis stigma. The patients experienced isolation within their family and community, separation, and financial crisis. The stigma attached to tuberculosis may contribute to delayed healthcare seeking, poor treatment adherence, and poor prognosis.

**Conclusion:**

Interventions thatreduce the stigma attached to tuberculosis should target on areas, such as creating community awareness, patient counseling on problem-solving and emotional skills, preparing culturally sensitive and scientifically sound media messages, providing financial support for the patients, and enhancing the qualities of the healthcare workers, such as empathy, concern, respect for the patient and cultural sensitivity.

## Background

Tuberculosis (TB) is a social and biological illness of humankind that causes significant challenges to public health worldwide [1]. Stigma is one of the major obstacles to TB control [2–4]. It is a social process that begins when a particular trait of an individual is identified as being undesirable or disvalued [5]. The stigmatized individual often internalizes this sense of disvalue and adopts a set of self-regarding attitudes about the trait including shame, disgust, and guilt [6]. These attitudes produce a set of behaviors that include hiding the stigmatized trait, withdrawing from interpersonal relationships, or increasing risky behavior [7, 8]. Similarly, stigma may lead someone with TB to hide symptoms, avoid or delay seeking care, conceal a diagnosis or default from treatment [9–11], and it can also affect care provided by family [12].These dangerous coping mechanisms contribute to sustained community transmission of the disease and the emergence of drug resistance strains.

Researches from high-burden areas have shown that TB patients face various levels of isolation and rejection from families and communities, including loss of employment,reduced education opportunities, vulnerability to disability, divorce or spoiled marriage prospects,and isolation at home that forbids sharing food, utensils or sleeping space [13–17]. Stigma attached to TB is caused by the severity of the illness, fear of infection andmyths about its transmission, prejudice, lack of access to services, ignorance,irresponsible media reporting, and by being confused with Human Immunodeficiency Virus (HIV) which is associated with perceived sexual misconduct [2, 3, 17, 18].

The Government of Ethiopia initiated a pilot TB control project based on the Directly Observed Treatment, Short-course (DOTS) strategy in 1992. Since then the program has been subsequently scaled up in the country and has reached 100% geographic coverage according to the 2008 Federal Ministry of Health report [19]. However, the country remains among high TB endemic countries in the world with annual incidence and prevalence of 207 and 200 per 100,000 people, respectively. The case detection rate of the country was 60% [1].

Stereotypic conception of TB as a threat to society conveys a devalued social identity about the patients, and underlies the beliefs, thoughts, and economic and political actions of the nation when interacting with the patients [20]. This may influence the patients’ desire to seek medical help [21].

In spite of the recognition that TB stigma has a negative effect on TB patients and the control programs, its form is not well understood in the context of Ethiopia where people encounter multiple socio-cultural barriers that interplay between health and illness. This can lead to inappropriate stigma-reduction interventions being developed which at best do not work and at worst may even increase stigma. In response to this, this study explored the causes and effects of stigma against TB patients at St. Peter TB Specialized Hospital in Addis Ababa, Ethiopia. The study findings will help to institute appropriate interventions to minimize the impact of stigma on sufferers and TB control programs. It also provides a rigorous descriptive base upon which subsequent explanatory research can be based.

## Methods

### Study Area

The study was conducted at St. Peter TB Specialized Hospital in Addis Ababa, Ethiopia. The hospital was established in 1961. It is a governmental hospital run under Federal Democratic Republic of Ethiopia- Ministry of Health. The hospital provides TB diagnostic, treatment and care services.

### Study Design

A phenomenological study design of qualitative methodology was used.Phenomenological researchis a type of approach in which the researcher identifies the human experiences concerning a phenomenon, as described by participants in a study. Understanding the lived experiences marks phenomenology as a philosophy as well as a method, and the procedure involves studying a small number of participants to understand patterns and relationships of meaning. In this process, the researcher works to take the experiences of participants on the participants’ own terms. The approach was chosen as a relevant design to explore the causes and effects of stigma against TB patients [22].

### Theoretical Framework

This research was centered on understanding of stigma attached to TB patients in relation to their social context. Following this, a phenomenological approach to research conceptualization, implementation and analysis was applied. The theoretical tenets of constructivism-interpretivism were appropriate for this frame of reference. Constructivism-interpretivism holds that reality or truthis framed within an individual’s experience rather than an external objective entity. The individual is thus the expertsource of information for that experience. Further, reality is shaped by the social milieu within which individuals exist. That is, constructions of reality are not made in isolation but against a backdrop of people’s historical and sociocultural contexts [23].

### Study Population

Purposive sampling encourages detecting cases within extreme situations [24, 25]. It allows for the capture of detailed descriptions of all aspects of the social phenomena or cases under study as a means to document the uniqueness of particular cases and instances; and, also to allow for the capture of important shared or latent patterns that may cut across cases in spite of their heterogeneity [25].

The health personnel in charge of TB patients in the selected hospital were contacted and asked to select TB patients for in-depth interviews using the TB register book. Because considerable experience of living with TB was considered very fundamental for effective deliberation on stigma experience, only patients who had had at least two months of treatment were recruited to participate in the study. Healthcare workers (HCW) who were providing care for the patients at the hospital during the time of interview were selected for key-informant interviews.

### Interviews

Interviews were carried out by the investigator between July and August 2015. All participants were interviewed face-to-face, on one occasion only, in private and on-site, and sessions were audio-recorded. The majority of interviews were recorded using a digital recorder. Notes were taken during interviews to capture emotions expressed verbally or non-verbally, and in cases where the respondents did not wish to be recorded and expanded immediately afterwards. Interview-guide questions prepared in Amharic (local language of respondents) were used for both in-depth and key-informant interviews. The questions were not rigidly adhered to, but served as a guide for a structured conversation and to ensure all topics were covered. While data collection was ongoing, preliminary analysis of the early interviews provided a means to adapt questions to the lived reality of participants’ experiences, refine probes, and to help note when an adequate sample size was reached. The researcher built a relationship of trust with the study participants to encourage them express their thoughts freely, and talk about their understandings of social circumstances in their own terms. Next, patients were asked questions that tapped into how they spoke about their illness to people within their social networks, their experiences in their home and communities, and their experiences accessing healthcare, such astheir health-seeking behavior before ending at the hospital. HCW were asked to share their thoughts about TB based on their day-to-day experiences providing care. Probes were used to ascertain the meaning of participants’ accounts, clarify a point, open new themes for discussion, or encourage further elaboration. All interviews took 1 to 1.5 hours.

### Data Analysis

Qualitative analysis is a highly fluid process involving non-linear movements back and forth between field notes, transcripts, personal thoughts, and emerging ideas. The investigator transcribed the audio-records on the same day as the completion of the interviews enabling him to capture observations of the non-verbal points by linking the audio-recorded interviews, field notes, and the researcher’s memory of the event. The transcriptions were done in an undisturbed environment. The researcher embarked on the following steps: 1) each recorded interview was downloaded into the researcher’s laptop, 2) each folder was allocated a unique file name containing the date of the interview for identification purposes, 3) recorded interviews were downloaded to disks creating a backup copy, 4) a verbatim translation of the transcripts from Amharic into English was done by the researcher. The translation was checked by listening to the recorded interviews again whilst reading the computer files, 5) after completing the transcriptions, computer folders, downloaded disks, and transcribed and translated notes were labeled using identification number, date and place of interview to make connection back at any time when the researcher is in need, and to ensure confidentiality. Each transcription took 1–2 hour(s).

The researcher immersed himself into the data by repeatedly hearing of audios and reading of the transcribed and translated notes. He used the Open Code qualitative data analysis computer software package to analyze the data. The decision to use this package was based on the nature of the research topic: exploration of the causes of TB stigma and its effects on TB patients and TB control programs.Coding of the data was started immediately after the translation of the data to avoid memory loss as time goes on. The coded data were further categorized. That means the codes with similar characteristics were grouped together thematically.

Events or experiences that contributed to a set of themes were set apart from those deviating from established patterns. In other words, negative cases were equally drawn out and analyzed. Why was it that most patients appeared to have certain common experiences but one patient experienced something sharply contrasting? What led to this markedly different construction of events, and interpretation of the same? Attention was paid to unexpected themes or surprise cases and even non-themes. Discovery of these findings and further analyses of them was recursive, involving moving back and forth between different levels of coding and conceptualization.

The data analyses and interpretation happened simultaneously. The researcher provided interpretation based on the findings to increase the transferability of the study to other context. In addition, cases were drawn from the interviews to explain specific stories in detail. And then the preliminary findings were presented to colleagues to receive input and comments. Finally, data which show the process, records, documents and findings were kept for audit trail.

### Ethical Considerations

The study protocol and consent procedure were approved by School of Public Health, Addis Ababa University. Permission was obtained from office of the St. Peter TB Specialized Hospital. At the beginning of every data collection session, the purpose of the study was explained and verbal consent obtained from every participant. The consenting procedure was audio-recorded. Written consent was not obtained because of the continuous consenting process of qualitative design. Participants were assured that their participation in the interview was voluntary and that they could decline to answer any question or end the interview at any point without providing a reason. Patients were also assured that their decision to participate and individual responses would not affect their current or future medical care. Their responses would not be shared with site staff and other patients. No form of inducement to entice the participants to partake in the study was done. The anonymity of participants and confidentiality of the information were maintained throughout the study by using pseudo identification and removing personal identifiers. Presentation of a large section of data was avoided so as to prevent the possibility of readers identifying the person speaking in the quote.To help protect the identity of the patients interviews were held within the hospital premise. All the recorded and written data were kept in a secured place and that was explained to the study participants prior to interviews.The computer used for the data retrieval and analyses had only one entry and was password protected.

## Results

A total of 10 in-depth interviews were conducted with 5 female and 5 male TB patients, and 6 key-informant interviews with HCW. The average age of the patients was 33.7 ± 3.2 years. Except one patient nine of them attended only primary education, and had no formal employment. Five of the patients had extrapulmonary TB, three had multidrug resistant TB, and two had smear-positive pulmonary TB. Three ofthem were retreatment cases.

The in-depth and key-informant interview findings were combined, and presented in three themes. In theme one, the causes of TB stigma identified in the data were explored, whilst the effects of TB stigma on patients and TB control program in theme two and three, respectively.

### Theme One: Causes of TB Stigma

#### Fear of infection

The fear of infection was explored by the patients to be the cause of TB stigma. TB was described as a dangerous and highly infectious disease that spread through the air as well as by personal contact with individuals affected with the disease.

*“People stigmatize against me*. *I usually feel loneliness*. *The worst is when my relatives visit me they keep themselves at a distance and never approach me*. *Just they come and go*. *Actually*, *I can’t judge them as bad ones*. *They do so because they fear being infected*.*”*

A HCW witnessed this as:

*“People are not willing to approach TB patients…Even I remember a multidrug resistant TB patient returned to his home due to the fact that the HCW refused to treat him…TB is a fearful disease*.*”*

#### Inappropriate health education messages by media

Inappropriate health education messages by media, such as television and radio were mentioned as the basis of society’s poor attitudes and behaviors towards TB patients. A HCW clarified this as:

*“The media broadcast the TB messages irresponsibly*. *They focus only on the infectious nature of TB*, *and opening the windows as the only way to prevent TB transmission*. *This is incomplete message and unnecessarily creating exaggerated fear among the members of society*. *Nevertheless*, *the media should convey full pledged messages addressing the diagnosis*, *treatment and care and prevention of TB*.*”*

A TB patient explained this as:

*“What I learn from media (television and radio) is that TB is a contagious disease*. *It will be better if media address about the diagnosis and treatment and care of patients in detail so that the disease burden can be reduced in the future*. *Otherwisemedia continuesto be a sole source of stigma against TB…”*

### Theme Two: Effects of TB Stigma on Patients

#### Isolation within the family

The data revealed that patients experienced various forms of negative attitudes and behaviors from close and household contacts.

*“I suffered a lot*. *Besides the burden of the disease*, *I am separated from my family*. *I eat alone*, *sleep alone*, *and live alone*. *Nobody come close me*. *Moreover*, *they totally avoided me since I have been admitted at this hospital*.*”*

In contrast, a patient stated that he didn’t experience any form of isolation fromhisfamily members.

*“I have good families…They never leave me alone*.*”*

#### Isolation within the society

The community members established a desire to keep away from TB patients because they are conscious that association with them could result in infection.

*“…I live in a rented house*. *The house owners observed me sick with weight loss*, *coupled with a persistent cough*, *and sometimes coughing up blood*, *they felt very uncomfortable and banished me from their house*. *The next house owners did the same just after three days of my renting…”*

*“…When interacting with members of the society*, *the people either move away to stand at a distance or turn their heads in the opposite direction*, *probably to avoid being infected*.*”*

A HCW corroborated this by saying:

*“Always*, *we observe our patients outside the hospital not wearing masks*, *or evena handkerchief*. *Some patients do not also disclose their diagnosis of TB to their close mates…The patients do these because they fear being stigmatized*.*”*

#### Separation of TB patients from others

The disease also affected the way the patients related with others. Most (5 of 10) patients cut off themselves from dealings with others, and preferred living secluded lives just to avoid being stigmatized.

*“I don’t closely approach people since I have TB*. *Usually I prefer being alone…Why should I be stigmatized*?*”*

When it became unavoidable for them to interact with others, the patients said they took measures to avoid infecting them.

“*However people stigmatize me in various ways*, *I always ensure that I will not infect them by keeping my selves a distance*. *I have TB*, *why make others suffer*?*”*

Some (4 of 10) patients used diverse names when they came to the hospital or did not want anybody to see them.

*“…I lost my identity*. *I used to be named by a diverse name at this hospital*. *I do this because I don’t want anybody to see me as a TB patient…”*

#### Financial burden of TB

Although TB drugs are provided free of charge, becoming ill with TB can have a substantial financial impact on patients and households. TB causes physical weakness making it difficult to continue working or find work, treatment is lengthy and requires regular visits to a health facility requiring time off work and therefore lost income, and incurring travel expenses. The prior financial situation of a household, the opportunities for financial support, whether the patient has dependents and responsibilities, and the nature and flexibility of the person’s work determine the extent of the disruptiveness of their illness.

*“I am a widow*, *and have two children*. *Prior to becoming ill with TB*, *I used to feed myself and the kids by working as a daily laborer*. *After the illness I can’t go for work*, *and there is no any other means of earning a Birr*. *It has been one and a half year long since I have been admitted as an inpatient in this hospital*. *I have nobody who supports me…TB is financially crippling disease*.*”*

The patients also reflected that people around them were not willing to help them financially, thus aggravating the negative financial impact of having TB.

*“For dread of infection*, *people often failed to offer any prop up for me*. *This has further worsened the financial impact of having the disease*.*”*

In contrast, some (2 of 10) patients mentioned that their close contacts and friends were very supportive.

*“I don’t experience financial hurdles*. *My families*, *friends*, *and close mates usually provide me a financial support…”*

### Theme three: Impacts of TB Stigma on the TB Control Program

#### Delayed health seeking

The data indicated that most (5 of 10) patients with obvious symptoms visited for TB diagnostic after they spent long periods at their homes due to the stigma attached to TB in the society.

*“You can’t imagine how our society stigmatizes against TB patients*. *They do not even shake your hands; they isolate you*. *You believe me or not I stayed at my home without seeking diagnosis for about half a year though I had obvious symptoms evocative of TB…”*

Some (3 of 10)patients had taken various medications and that they did not tell the attending physicians that they had been having cough for long periods.

*“I had visited a number of health facilities and taken various medicines before I visited this hospital*. *I did not tell the attending physicians that I had been having cough for long periods*.*”*

#### Poor treatment adherence

Treatment adherence was known to be influenced by the attitude of families, friends and care provides, and the support patients received from them. A HCW explained this by stating:

*“The patients take a cocktail of drugs for a long period of time*. *In spite of the fact that they need to feed themselves with nutritious foods in order to counter side-effects of the drugs*, *they can’t do so because they can’t afford for the food from local markets*. *The nastiest is that people around them usually do not stretch their hands since they stigmatize against the patients*. *As a result of this*, *some patients are usually forced to terminate taking the drugs*.*”*

#### Poor prognosis

Processes of stigmatization can also lead to poor quality of life. A HCW corroborated this by saying:

*“Often*, *patients visited this hospital after they spent long periods in a very bad stateat their homes*. *The late initiation of treatment for these patients is less likely to improve the prognosis of the disease*. *Usually*, *the treatment outcome of such patients is either treatment failure or death*.*”*

## Discussion

The fear of infection was explored as the cause of TB stigma in this study. The fear of infection was often unjustified; this points to stigmatization of the disease rather than safety measures to avoid infection.Nevertheless, once a TB patient has appropriate treatment, he or she is no longer infectious after a few days of treatment. Family and community members avoided contact with the patients, with some really presenting anawkward posture alongside the continuing presence of TB patients probably to avoid being infected. The patients were also expected to employ separate tableware and serving dishes when eating. In most African societies sharing household activities, such as cooking and eating from a common bowl is the norm.The unjustified prohibition of such cultural norms and practices because of the disease can result in further isolation of the patients in society.In the same token, fragmented and poorly designed media messages were indicated as other hurdles leadingto fear of the disease in society. This may also add to the stigmatization of TB patients. Stigma is dependent on social, financial, and supporting powers [6, 26]. When media are broadcasting TB as a fearful contagious disease, it augments the fear of the ailment, and reinforces the stigma attached to the disease in society [27].

Isolation within family and society, and financial burden were the themes that emerged as effects of TB stigma on TB patients. Patients who managed to get to the hospital and were put on treatment had to endure a lot of psychosocial problems because of the stigma attached to the disease in society. The patients indicated that it was uncommon to have others sit near them or even shake their hands, probably to avoid being infected.The patients affirmed that they had to depend on communal support, such as monetary help, provision of foodstuff, and prayers from family unit and contacts as means of handling with the disease. However, the majority (8 of 10) said that for dread of infection people often failed to offer any prop up. Consequently, the patients interviewed generally described that they experienced financial problems.In Ethiopia, TB diagnosis and treatment are meant to be provided free of charge with the aim of decreasing the financial burden on patients [28]. However, the patients still experienced financial challenges that were related to transportation costs, medical examinations, hospitalization costs, and expenses of basic necessity. In addition, the needed ancillary treatment was charged with significant financial burden on patients [29]. These may have devastating consequences which can worsen the illness experience of those affected by the disease.

In this and previous studies delayed healthcare seeking, poor treatment adherence, and poor prognosis were indicated as impacts of TB stigma on TB control program [30–33]. Since stigma is socially constructed, the attributes that are stigmatizing are well known and shared in a culture [6, 30]. This means that the people may be aware of their stigmatizing approach and behaviors in the direction of TB patients. Consequently, those with symptoms suggestive of TB may be unsuccessful to come to the hospital. Because of the stigma attached to TB, patients frequently hide obvious signs and symptoms of the ailment, and give details as owing to non-stigmatizing circumstances, such as ordinary cold or malaria just to diminish the disdain of others [27, 34]. Moreover, this can affect their motivation to adhere to the long duration of TB treatment. Since non-adherence to treatment could be used as a strategy to relieve them from the pain of stigmatization, such societal attitudes and behaviors can lead to default from treatment [32, 33, 35]. Furthermore, this study revealed that most (5 of 10) patients reported to the hospital after they spent long periods at their homes, characteristically in a very awful state, due to the stigma attached to TB in the society. The late beginning of treatment makes successful treatment outcome less probable. Such delays in reporting to the hospital and the subsequent late initiation of treatment may account for the high mortality documented among TB patients in Africa [1]. The increased mortality from TB may habitually intensify the dread of the disease, and result in stigmatization of the patients in society.

This study was limited to people who were already accessing healthcare for TB. The voices of individuals who were not receiving any form of medical care were absent. Their inclusion might have allowed for a fuller understanding of the nuances of illness experience among people who are likely the most marginalized and most vulnerable to stigma.

## Conclusion

This study explored various causes of stigma attached to TB and its effects on patients and TB control programs.The fear of stigmatization makes individuals with very obvious signs and symptoms to attribute it to non-stigmatized diseases or hide the diagnosis from others. Those put on treatment may end up defaulting from treatment because of lack of support. These will contribute to sustained community transmission of the disease and the emergence of drug resistance strains.Interventions that reduce the stigma attached to TB should target on areas, such as creating community awareness, patient counseling on problem-solving and emotional skills, preparing culturally sensitive and scientifically sound media messages, providing financial support for the patients, and enhancing the qualities of the healthcare workers, such as empathy, concern, respect for the patient and cultural sensitivity.
